# Editorial: Synergizing large language models and computational intelligence for advanced robotic systems

**DOI:** 10.3389/frobt.2026.1882491

**Published:** 2026-06-15

**Authors:** Weiyong Si, Baoru Huang, Mien Van, Long Jin

**Affiliations:** 1 School of Computer Science and Electronic Engineering, University of Essex, Colchester, United Kingdom; 2 School of Computer Science and Informatics, University of Liverpool, Liverpool, United Kingdom; 3 School of Electronics, Electrical Engineering and Computer Science, Queen’s University Belfast, Belfast, United Kingdom; 4 School of Information Science and Engineering, Lanzhou University, Lanzhou, China

**Keywords:** computational intelligence, embodied AI, foundation models, human-robot interaction, large language models, natural language interfaces, robotics

## 1 Introduction

The convergence of foundation models, including Large Language Models (LLMs), Vision Language Action (VLA) models, with robotics and computational intelligence represents one of the most significant developments in modern AI research. Traditional robotic systems, while capable in structured environments, have long struggled with the safety, intuitive interaction, and adaptive reasoning required for real-world deployment. By integrating the natural language understanding and contextual reasoning capabilities of LLMs into robotic frameworks, researchers are now unlocking new possibilities spanning voice-driven manipulation, autonomous navigation, and human-robot interaction.

This Research Topic brings together five original research articles that explore the growing role of LLMs in robotic and intelligent systems. The contributions cover a broad range of applications, including supermarket assistance robots, bimanual teleoperation, home-care navigation, educational robots, and automated machine learning, but share a common focus: using the language and reasoning capabilities of LLMs to bridge the gap between human intention and robot action. These works offer new methodologies, system architectures, and empirical findings that advance our understanding of how LLMs and computational intelligence can be effectively used in advanced robotic systems.

## 2 Article summaries

### 2.1 Multi-level LLM interface for supermarket robots

Nandkumar and Peternel present a voice-based interaction system for supermarket robots. Four widely used ASR systems are evaluated across speaker gender and language, with OpenAI Whisper achieving the lowest error rates, and language found to be a more significant source of variation than gender. Building on this, the authors propose a hierarchical multi-LLM conversational agent where a fine-tuned DistilBERT classifier routes user queries to specialised GPT-3.5 Turbo models, grounded to a product database using Retrieval-Augmented Fine-Tuning to reduce hallucinations. When compared against a GPT-4 Turbo benchmark, the proposed system performs better across all evaluated criteria. The work also demonstrates integration with a mobile robot for autonomous shelf navigation, suggesting that coordinated ensembles of smaller, task-specific models can be a practical alternative to relying on a single large model.

### 2.2 LLM-driven interaction framework for bimanual teleoperation

Fei et al. propose the Bimanual Teleoperation LLM Assistant (BTLA), a framework that allows a single operator to control two robotic arms simultaneously by teleoperating one arm directly while issuing voice commands to direct a second assistive arm. The system uses GPT-3.5-turbo to interpret natural language instructions and select appropriate manipulation skills in real time. Experiments show significant improvements in both task coverage and success rate over solo teleoperation, alongside a notable reduction in operator mental workload. The work demonstrates how LLMs can serve as an intuitive co-pilot in complex manipulation scenarios, removing the need for rigid pre-programmed commands.

### 2.3 LLM-integrated AI agent framework for intuitive home-care robot navigation

Xue et al. address the challenge of deploying LLM-powered robots on resource-constrained hardware, motivated by the growing demand for home-care solutions for the elderly. The proposed framework separates the robot’s pipeline into three modules: EnvNet for environmental perception, AIBrain for natural language understanding and planning using a quantized and locally deployed Llama two model, and RoutePlanner for navigation execution. The modular design is a deliberate choice, making each stage of decision-making independently interpretable and diagnosable. Results show promising navigation accuracy across a range of home environments, and the work provides a practical blueprint for deploying LLM-based agents on affordable, real-world robotic hardware.

### 2.4 Empathetic humanoid robot tutor via emotional intelligence, memory, and gesture integration

Sun et al. address a gap in educational robotics: while prior work has examined emotional intelligence, memory-driven personalisation, and gestural communication individually, their coordinated integration into a working system had not been fully explored. This work builds a robot tutor around Meta’s Llama 3.2 multimodal LLM, augmented with three modules covering emotion recognition, student memory and recall, and gesture synchronisation. An Engagement Vector Model is introduced to measure student engagement across cognitive, emotional, and behavioural dimensions. Results across three progressive trials show that the fully integrated system consistently outperforms the baseline robot, with improvements in engagement scores, quiz performance, and task completion times. The study demonstrates that robot tutors with empathetic capabilities can create a more supportive, interactive learning experience that ultimately leads to better outcomes for the student.

### 2.5 LLM-driven AutoML from a human-centered perspective

Yao et al. examine how LLMs can lower the barrier to machine learning by allowing users to build and run deep learning pipelines through natural language conversation alone. The system, built on the AutoM3L framework, is evaluated through a user study comparing an LLM-based interface against conventional AutoML programming across participants with varying technical backgrounds. Results show clear improvements in task completion rates, accuracy, and efficiency in favour of the LLM condition, with participants also reporting reduced complexity and faster learning curves. The study provides practical evidence that natural language interfaces can make machine learning more accessible across different levels of expertise, though limitations remain for highly specialised tasks.

## 3 Conclusion

The five articles in this Research Topic collectively demonstrate that LLMs are not merely text generation tools but effective interfaces that enable robots and intelligent systems to understand human intent, adapt to individual users, and perform complex tasks across diverse real-world settings. Key challenges remain, including reliability in grounded tasks, computational cost on resource-limited hardware, and the evaluation of long-term human-robot interaction. Future work should explore multimodal LLMs that integrate vision and language in real time, as well as longitudinal studies on sustained human-robot relationships. We thank all contributing authors, reviewers, and editors for their efforts, and hope this Research Topic will inspire further innovation at the convergence of language, intelligence, and embodied robotic systems.

